# Medical Students’ Perceptions and Use of Formal and Informal Curriculum Resources

**DOI:** 10.7759/cureus.52454

**Published:** 2024-01-17

**Authors:** Reanne Mathai, Sahil Patel, Emily R Littman, Angela Lo, Benjamin Yitzhak, Atsusi Hirumi

**Affiliations:** 1 College of Medicine, University of Central Florida, Orlando, USA; 2 Medical Education, University of Central Florida, Orlando, USA

**Keywords:** curriculum, pre-clinical medical education, motivation, learning, medical education

## Abstract

Introduction

Resource overload describes the feeling medical students experience in choosing formal (faculty-prescribed) and informal study resources (not faculty-prescribed). This study aims to characterize students’ use and perceptions of formal and informal study resources to inform their use in medical education.

Methods

This is a mixed-methods study utilizing a convenience sample of first-year medical students enrolled at the University of Central Florida College of Medicine during the academic year 2020-2021. A 40-question, five-point Likert scale, survey based on Keller’s Attention, Relevance, Confidence, and Satisfaction (ARCS) Model of Motivational Design was distributed to medical students during the end of their first year of medical school. Multivariate analysis of variance determined differences between formal and informal resources for each construct. Interviews were also conducted by first-year medical students and analyzed using thematic analysis. Learning logs were completed during the beginning of the medical students' second year to assess daily study habits.

Results

Fifty-one students completed the survey with a response rate of 42.5%. Informal resources scored higher across all constructs: attention (formal: 3.4±1.2, informal: 4.0±1.1; p<.0125), relevance (formal: 3.8±1.1, informal: 4.3±1.0; p<.0125), confidence (formal: 3.2±1.2, informal: 4.1±1.1; p<.0125), satisfaction (formal: 2.8±1.2, informal: 3.6±1.2; p<.0125) (Likert scale 1-5, Mean±SD). Students found formal resources lacked depth and organization while informal resources allowed for concise understanding with retention cues. Learning log data reported similar use of formal and informal resources during week 1 (88.2% formal vs. 87.8% informal) and week 2 (84.6% formal vs. 82.6% informal).

Conclusions

Students preferred informal resources based on ARCS constructs. However, the actual usage of formal and informal resources was similar. Formal resources align more with curricular assessments, but informal resources aid student retention and understanding. Therefore, students find both formal and informal resources necessary for success. Faculty should consider integrating informal curriculum resources to optimize student learning.

## Introduction

Historically, the United States Medical Licensing Examination (USMLE) Step 1 board licensing examination has been considered one of the most important assessments in a medical student’s early career. Based on the didactic information students receive during their first two years of medical school, medical students taking the USMLE Step 1 examination used to receive a three-digit score with a passing score of at least 194, with an average score of 231 nationally in 2021 [1,2]. However, beginning on January 26, 2022, the USMLE Step 1 shifted to pass/fail scoring, with a slightly higher passing score of 196 [3]. Even with this change, failing STEP 1 significantly decreases a medical student’s chance of matching into the residency of their choice. According to the National Resident Match Program (NRMP) Program Director Survey, when it came to deciding which applicants to interview for available residency positions, any failed attempt at a USMLE examination had the greatest negative influence, with an importance factor of 4.4 out of 5 [4]. As a result, most medical students prepare for the USMLE Step 1 examination early in their medical school education using formal and/or informal resources [5].

Formal resources refer to curriculum resources that have been recommended by medical education faculty. This includes faculty lectures, PowerPoint slides (Microsoft Corporation, Redmond, Washington, United States), self-learning modules (SLMs), recommended textbooks, and in-house practice questions and cases. On the other hand, informal resources refer to all resources recommended by peers and not prescribed by faculty. Informal resources include pre-made flashcard decks, supplemental videos, question banks, self or peer-created study materials, and other commercial third-party resources.

Recently, there has been an exponential increase in the number of commercial third-party resources available to help students prepare for USMLE Step 1. Many of these informal resources have been created with proven study strategies, including spaced repetition and testing [6-14]. For example, UWorld is a question bank of over 3,600 questions similar to those asked on USMLE Step 1. In addition to the questions, UWorld aims to simulate the testing environment and give students in-depth explanations for each correct and incorrect answer [12,13,15]. Additionally, Anki is a computer application that utilizes a pre-programmed schedule to allow for spaced practice of material, either through the use of pre-made flashcard decks or through flashcards created by the user [6].

Furthermore, although not based on spaced repetition or testing, First Aid for the USMLE Step 1 is a review book that aims to help students identify “high-yield” concepts for the test [16]. The use of UWorld, Anki, and First Aid have all been correlated to higher licensing examination scores [6,11,17-19]. A study conducted by Deng et al. demonstrated that each additional 445 board-style practice questions or 1700 unique Anki flashcards were associated with an additional point on Step 1 when controlling for other academic and psychological factors [6].

When added to the extensive number of formal resources prescribed by faculty, the expansion of third-party resources has left students feeling inundated by the plethora of study materials to choose from. Currently, little research has been done on how medical students choose and utilize these resources, which is important to medical schools when they are creating their educational curriculum and incorporating effective study resources. Even when schools provide newer commercial resources, such as Pathoma (Pathoma LLC, Lincolnwood, Illinois, United States), SketchyMedical (Sketchy Group, LLC, Los Angeles, California, United States), and Boards and Beyond (B&B) (Ryan Medical Education, LLC, West Hartford, Connecticut, United States), the effectiveness of the resource is rarely evaluated to guide future practice [20]. This study aims to analyze medical students’ use and perceptions of both formal and informal resources in their pre-clinical years which helps in preparation for the USMLE Step 1 licensing examination.

## Materials and methods

Design

This is a mixed-methods study utilizing a convenience sample of students who voluntarily participated. The study consisted of the use of a survey, interviews, and learning logs to evaluate formal versus informal resource utilization for learning among medical students. First-year medical students completed both the survey and interview during the academic year 2020-2021. When these students started their second year of medical school during the academic year 2021-2022, they then completed the learning logs.

Participants

First-year medical students enrolled in the University of Central Florida College of Medicine (UCF COM) during the academic year 2020-2021 participated in the study. A written waiver of consent was completed by each participant for data collection. Institutional Review Board approval was obtained through the UCF COM (approval number: STUDY00002339, dated December 18, 2020). Each participant received a total of $30 in gift cards (two learning logs and one survey). We received a $6,000 grant for gift card use from the University of Central Florida Medical Education Department.

Inclusion/exclusion criteria

First-year medical students enrolled at UCF COM during the academic year 2020-2021 who consented to participate and had access to email were included in this study. Those who did not have access to email, were not able to read or speak English, or who chose not to participate were excluded.

Likert scale survey

The survey, interview questions, and learning logs were created by the research team. A 40-item, five-point Likert scale, Qualtrics questionnaire (Qualtrics International Inc., Seattle, Washington, United States) was adopted by the researchers based on Keller’s Attention, Relevance, Confidence, and Satisfaction (ARCS) Model of Motivational Design as an indicator of their motivation to use formal and informal resources. The questionnaire measured students’ perceived levels of ARCS with conventional and commercial resources recommended by classmates. Each of the four constructs was allotted 10 items on the questionnaire, five questions regarding conventional resources and five questions regarding commercial resources. The Likert scale ranged from 1 to 5, with a response of “5” indicating that the curriculum resource was very effective in that specific construct and a response of a “1” indicating that the resource was very ineffective. Cognitive pretesting was not completed prior to administration of the questionnaire. The survey was distributed to 120 first-year medical students. Participation was voluntary and each student received a $10 gift card upon survey completion.

Ethnographic interviews

Individual interviews with first-year medical students were conducted to elaborate on survey data and better understand students’ resource use and perceptions. One-hour individual interviews were completed on a virtual platform and consisted of open-ended questions. Each student was asked the same open-ended questions, which included asking about the types of formal and informal resources the student uses to study and their process of using the resource. Two independent coders analyzed the interviews using thematic analysis to ensure interrater reliability and an in-depth understanding of the students’ experiences with study materials.

Learning logs

To assess daily study habits, learning logs were distributed for daily completion to all study participants. Distribution occurred daily for one week during two different modules. Participants completed the learning logs at the end of each day, tracking the use of both formal and informal study resources along with the duration of use. Participants received a $10 gift card upon completion of the learning logs for each week.

The learning logs were distributed once medical students started their second year of medical school (academic year 2021-2022). The learning logs were completed by the same medical student class who completed the surveys and interviews. Participants completed two separate one-week-long learning logs for use during different modules to increase the quantity of data and decrease the potential bias of gathering all data during a single time frame. During the first learning log collection, 229 entries were made with 235 entries in the second learning log. Attempts were made to retain the same participants during both weeks. The learning logs were administered through Google Forms (Google LLC, Mountain View, California, United States). Each learning log was subdivided into formal and informal sections. The students were asked to divide the total time spent studying that day between formal and informal resources and then log the quantity of time spent on each resource.

Data collection

An electronic link for survey and learning log completion was sent to participants via email daily and completed anonymously. Participants completed the learning log in their leisure time, upon conclusion of their daily studying. Survey collection was managed through Google Forms.

Statistical analysis

Multivariate analysis of variance was used to determine differences between formal and informal resources for each construct to analyze the survey results. To reduce the probability of a Type 1 error, Bonferroni adjustment was utilized; statistical significance was determined at p<0.0125. Psychometric testing was also conducted to ensure survey reliability. Two independent coders analyzed the interviews using thematic analysis to ensure inter-rater reliability and an in-depth understanding of the students’ experiences with study materials. Data analysis was run on the learning logs to determine the proportion of students who utilized each study resource.

## Results

Likert scale survey

**Figure 1:**
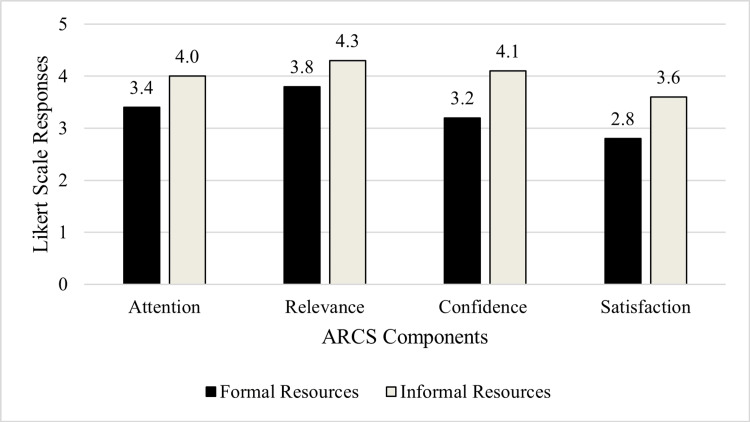
Student Perceptions of Formal and Informal Curriculum Resources Using the ARCS Model. ARCS: Attention, Relevance, Confidence, and Satisfaction

Of 120 first-year medical students, 51 (42.5%) completed the Likert scale survey. Cronbach's alpha was greater than 0.7 for each subscale. Students rated informal teaching methods higher across all ARCS constructs: attention (formal: 3.4±1.2, informal: 4.0±1.1; p<0.0125), relevance (formal: 3.8±1.1, informal: 4.3±1.0; p<0.0125), confidence (formal: 3.2±1.2, informal: 4.1±1.1; p<0.0125), satisfaction (formal: 2.8±1.2, informal: 3.6±1.2; p<0.0125) (Mean±SD) (Figure 1).

Thematic analysis

**Figure 2:**
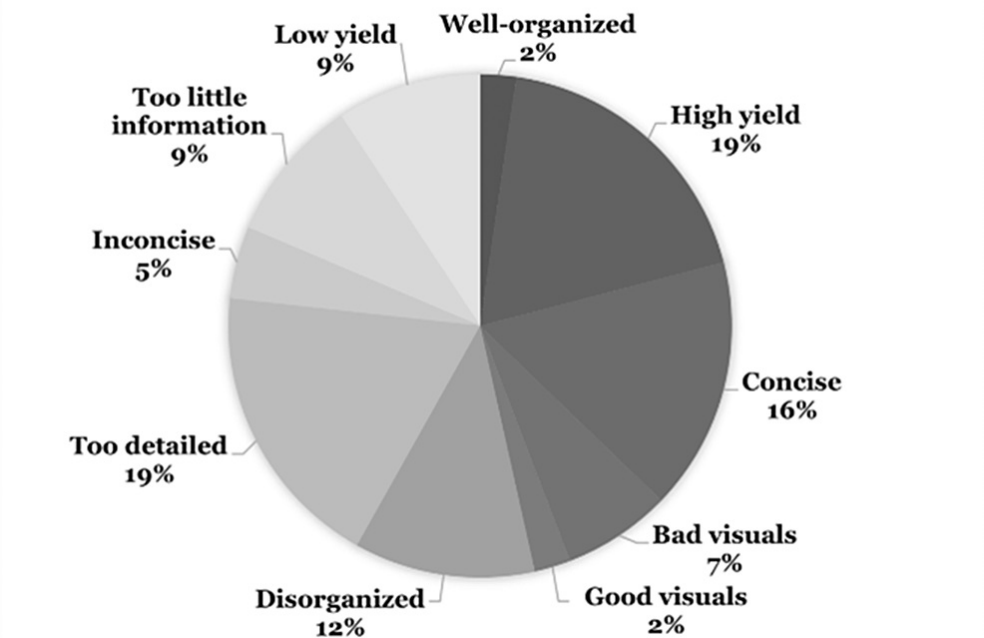
Student Perceptions of Formal Curriculum Resources.

**Figure 3:**
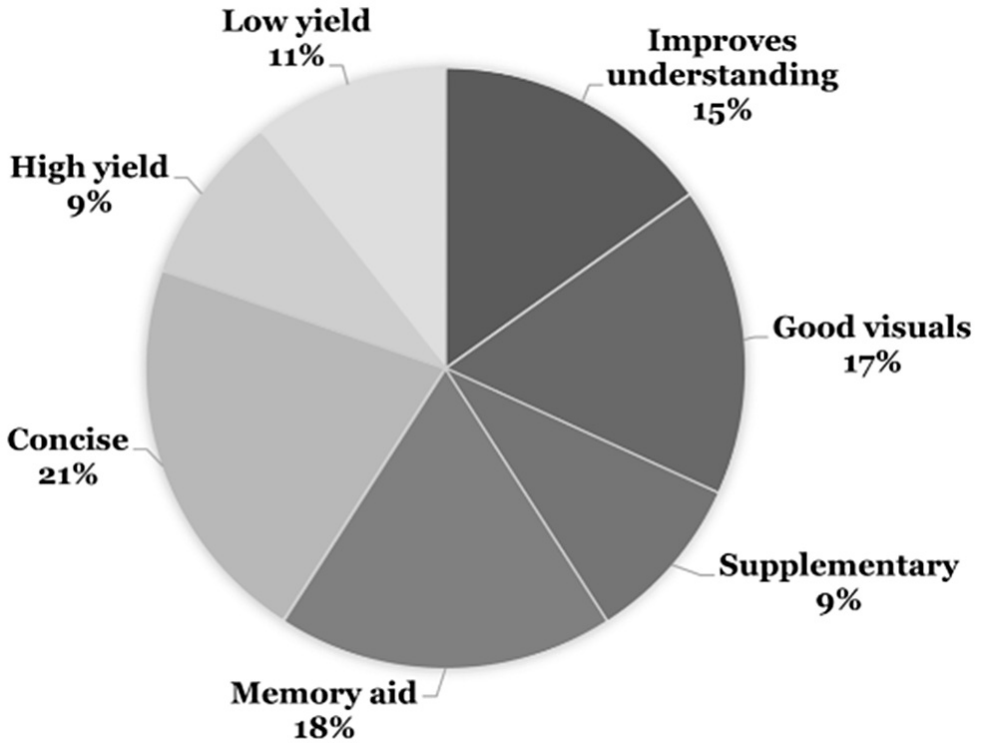
Student Perceptions of Informal Curriculum Resources.

Researchers conducted thematic analysis interviews with the students, utilizing the record and transcription features of Zoom (Zoom Video Communications, Inc., San Jose, California, United States). After the interviews, each recording was independently analyzed by two researchers to extract the overarching themes of the initial 21 interviews conducted (Figures 2, 3). 

Learning logs

**Figure 4:**
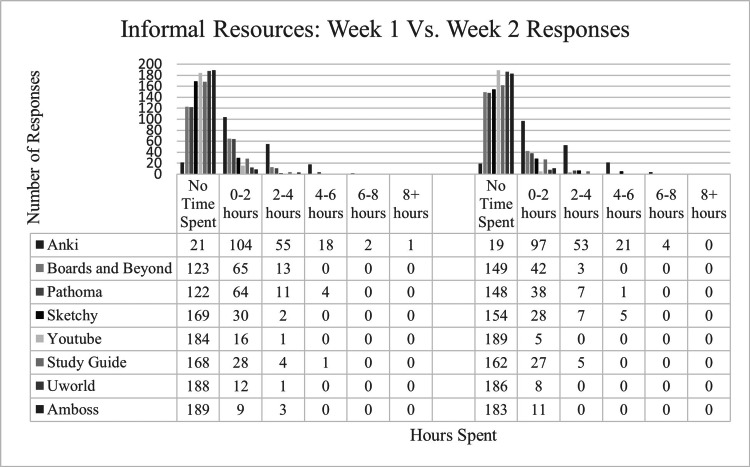
Student Time Spent on Various Informal Curriculum Resources.

**Figure 5:**
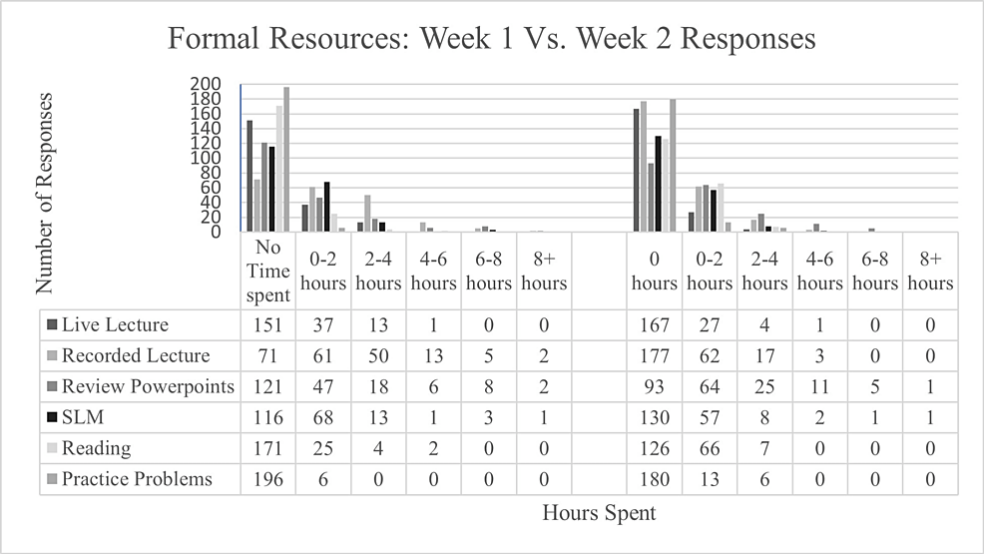
Student Time Spent on Various Formal Curriculum Resources. SLM: self-learning module

During the first week of learning log data collection, 229 responses from 33 second-year medical students were recorded. Of these responses, 202 (88.2%) were reported by students using formal resources and 201 (87.8%) indicated that students used informal resources. The time spent utilizing each resource by survey participants is outlined in Figures 4, 5. In addition, students indicated the following formal resources not covered in the survey: Clinical pre-session readings (CPR) grids (n=4), team-based learning (TBL) cases (n=5), clinical skills videos (n=2), suture clinics (n=1), physical examination practice (n=2), and history and physical (H&P) writing practice (n=1). Students also indicated utilizing the following informal resources not covered in the survey: Goljan lectures (n=1), physiology videos (n=1), Osmosis (n=1), Picmonic (n=1), and Lecturio (n=1). 

During the second week, the survey received 235 responses from 33 students. Of those, 199 (84.6%) students indicated that they used formal resources, and 194 (82.6%) students indicated that they used informal resources. In addition, students indicated the use of the following formal resources not covered in the survey: TBLs, Physical Exam Teaching Associates (PETA) sessions and clinical skills examination prep, curriculum-mandated research project, and peer-directed exam review. Students also indicated the following informal resources not covered in the survey: First Aid, Study Group, and UpToDate research articles. 

Students were also asked to indicate how their study habits have changed over time. The responses were as follows for week 1: “Changes daily” (n=5), “Less Anki” (n=3), “More third-party videos for review” (n=3), “More premade Anki from classmates” (n=1), “More lectures - third party info gap” (n=1), “Less in-person lecture” (n=1), “More YouTube - help with clinical questions” (n=1), “Watching outside resources before formal” (n=1), “More Anki in general” (n=1), and “More practice problems” (n=1).

The responses were as follows for week 2: “Changes daily” (n=5), “More focus on class material - specifically lectures and PowerPoints” (n=3), “More premade Anki decks with class material” (n=3), “Supplement with B&B and Pathoma” (n=3), “Balance formal and informal” (n=1), and “Studied more before TBL” (n=1).

## Discussion

According to a literature review conducted by McGaghie, the validity and support of the commercial courses on medical examination performance are weak or nonexistent, suggesting that there is little research on commercial courses in medical education [21]. However, studies that have been conducted correlate with the findings of the present study. For example, a literature review conducted by Hirumi et al. showed a positive correlation between medical student use of commercial question banks and scores on licensing board examinations [5]. Specifically, a study of Tulane students showed that doing more than 2,000 practice questions correlates with higher scores [22]. Similar to the present study, although conducted prior to the coronavirus disease 2019 (COVID-19) pandemic, it calls for the incorporation of commercial resources into conventional curriculum resources to aid students in preparation for board examinations and to reduce the cost of these beneficial resources. This is additionally supported by a survey study conducted by Giordano which indicates that the top two resources for STEP 1 examination preparation were both commercial resources (First Aid and UWorld) [13]. Additionally, in a self-directed survey of 500 faculty members in South Korea on the perception of e-resources, 80 participants responded and the consensus was that though e-resources were helpful, students either felt they didn’t have time to use them, couldn’t find relevant resources, or didn’t know they were available to use [23]. These concerns were similar to those found in our study. Incorporation of commercial resources into the conventional curriculum would aid in students’ confusion about how to use and locate these resources. Incorporating commercial curriculum resources into conventional resources would also maximize student study time to decrease burnout. According to the study conducted by Giordano, the number of study days did not have a correlation with scores, suggesting that increased study time may not ameliorate poor grades [13]. Rather, the types of resources used, and the information covered during the study period likely matter more. Studying for 8-11 hours per day and studying for less than 40 days was correlated with higher scores [22]. Therefore, it is best to encourage students to use resources that maximize this limited timespan, including those with high scores in the ARCS constructs.

Likert scale survey and thematic analysis

By the end of their second module, first-year medical students reported a significantly greater level of overall motivation to learn from informal vs. formal resources on the Likert Scale survey. Specifically, students rated informal resources significantly higher across all ARCS themes compared to formal resources. Thematic analysis of the interviews suggests students found that informal resources allowed for stronger and more complete understanding, had clear and concise presentations, and provided cues to improve retention, while formal resources lacked depth, organization, and clarity.

Additionally, students viewed informal resources as higher yield for board examinations. However, they deemed formal resources higher yield for in-house curriculum examinations. During the interviews, students used the term “high-yield” when referring to formal resources and how well the information presented pertained to in-house curriculum exams. In contrast, students described informal resources as “high-yield” as they pertained to board exam preparation. Given that the interview data was gathered at the end of the first year, board exam preparation was unlikely to be the highest priority for students, which could explain why the “high yield” ratings were lower for informal resources at the time. In addition, board preparation was unlikely to have been fruitful without any exposure to content covered in the second year. Our findings compared well with Wynter et al. who found that the majority of students reported using online teaching videos (92%) and question banks (91%) [24]. However, when students were learning new material, most preferred reading textbooks and making written notes. On the other hand, when students were using resources for the revision of learned material, they most frequently used online question banks [24]. 

Learning logs: formal live lectures, recorded lectures, and PowerPoint slides

Between the two rounds of data collection among second-year medical students, usage of formal resources was roughly the same in weeks 1 and 2. Most students did not attend live lectures during both weeks of data collection, but they indicated using recorded lectures for zero to four hours on at least one day of the week. This is likely because they found recorded lectures to be more convenient, as they could view them at any time. They likely also preferred the ability to speed up or slow down the pace of the lecture. Recorded lectures also enable students to fast forward or rewind to certain parts of the lecture, which helps to explain this discrepancy as well.

Additionally, more students reported using PowerPoint slides to study in week 2 compared to week 1. As the academic year progressed, students might have found that in-house curricular exams included more material specific to faculty lectures, which may have encouraged them to take more time to review the PowerPoint slides later in the year. Study behaviors are influenced heavily by students’ perceived effectiveness of content delivery and peer recommendations. For this reason, students may opt to incorporate lectures into their studying during one module but not during another (Cardiology and Pulmonology versus Gastrointestinal and Renal, in this case).

Learning logs: formal SLMs, textbooks, and practice problems

Most students did not utilize SLMs for studying, and those who did only spent between zero to four hours doing so. This could be partially explained by delayed completion as most SLMs do not have a fixed deadline for completion. SLMs also tend to be shorter than full faculty lectures, which could also explain the low time spent utilizing this resource. Additionally, the vast majority of students denied using textbooks and recommended readings to study. One possible explanation for this is that textbooks may not be aligned with the learning objectives of the modules and may be too detailed for curricular and board exams. Additionally, the quality of resources recommended by module faculty could be different based on which module students are studying for.

In terms of formally assigned practice problems, although most students did not report using these resources to study, it is likely that they just had not used them during the time of data collection. Similar to SLM usage, the absence of a fixed deadline for the completion of formative quizzes could explain the low usage for that week. In addition, many students choose to complete the formative quizzes later in the module after feeling more comfortable with the material and as a study aid for the curricular exam, as these questions model those seen on the final exam.

Learning logs: informal resources

In terms of informal resources, Anki was the most heavily used resource among medical students in both rounds of data gathering. Students likely benefitted from the spaced repetition offered by this resource [6,19]. Upperclassmen also often made pre-made Anki decks, which encouraged use as a study resource as well.

In terms of lecture-style informal resources, more students preferred B&B and Pathoma over YouTube. The organized and clearly labeled videos of B&B and Pathoma compared to the inconsistent availability of YouTube videos on a given subject may explain this preference. SketchyMedical was reported as less used during the time of data collection. SketchyMedical is most often used for microbiology, pharmacology, and certain common pathologies [25]. However, if this material was not covered in the curriculum during the time of data collection, this may explain why a majority of students did not use this resource during these two weeks.

Most students denied the use of study guides, although they may have utilized this resource more frequently closer to the exam date. The informal resources that were used the least were question banks such as UWorld and Amboss. Given that such question banks are mainly used as preparation for national board exams and STEP 1 transitioned to pass/fail grading, fewer students utilized these question banks as a study aid for curricular exams.

Limitations

This study is only representative of a single Southeastern public institution in the United States, which may limit the generalizability of our results. However, it is suggested that students across the nation have similar study habits and use similar resources [11]. Interviews were conducted at the end of the first year and not during the second year, so changes in perceptions and usage of resources were not documented. In addition, learning logs represent just two weeks of the second year, which is unlikely to account for all the variability in study habits among different modules. This is especially true as students’ study behaviors often change as an exam date approaches. As such, students would have likely not participated in the learning logs during this time, as it may be seen as a distraction to their studying.

## Conclusions

Although most students indicated a preference for informal resources based on ARCS constructs at the end of their first year, the usage of both formal and informal resources was similar in the second year. Despite preferring informal resources to formal ones, formal resources tend to align more with curricular assessments (TBLs, quizzes, module exams, and any other graded assignments). This alignment might motivate students to use formal resources. In contrast, informal resources help students to retain and understand information taught in the classroom and provide a stronger foundation of knowledge. As such, the usage of both formal and informal resources is essential to the success of the average medical student for both curricular and board examinations. Faculty should consider integrating the use of informal curriculum resources to optimize time spent on preparing and delivering information. More research is warranted to determine how students use and perceptions may change over time.

Additional student learning logs and interviews of students and faculty are planned to determine a collective perception of the most effective study resources. The same methods and data were collected for second-, third-, and fourth-year students as well as faculty, which will be used to triangulate the findings of this study for future curriculum innovation. In addition, future studies are planned at other medical schools throughout the country to better understand the average medical student’s study habits and preferred study resources.
